# Evaluation of the Acute Basic Palliation Concept by Relatives and Health Care Professionals: An Observational Study of 40 Home-Dying Patients in Denmark

**DOI:** 10.1089/pmr.2024.0062

**Published:** 2025-02-05

**Authors:** Dorte Melgaard, Mike B. Astorp, Johannes Riis, Inez Madeleine Jensen, Anne Louise Hartvig Skalborg, Matilde Alida Arendt Eriksen, Camilla Ly, Bensu Izgi, Line Elise Møller Hansen, Anne Lund Krarup

**Affiliations:** ^1^ Department of Emergency Medicine and Trauma Care, Aalborg University Hospital, Aalborg, Denmark.; ^2^ Faculty of Clinical Medicine, Aalborg University, Aalborg, Denmark.; ^3^ Department of Emergency Medicine, North Denmark Regional Hospital, Aalborg, Denmark.; ^4^ Department of Geriatric Medicine, Aalborg University Hospital, Aalborg, Denmark.

**Keywords:** acute medicine, discharge, end-of-life care, emergency medicine, municipality, palliation, terminal care

## Abstract

***Background:*** Many individuals prefer to pass away in the comfort of their own homes, yet logistical obstacles often result in their admission to hospitals for end-of-life care.

***Objectives:*** To measure the effectiveness, as assessed by relatives and staff, of end-of-life care according to the acute basic palliation concept (ABPC) for patients discharged from an emergency department.

***Methods:*** An observational study of 40 consecutive actively dying patients who were discharged from Aalborg University Hospital, Denmark, using the ABPC. Effectiveness of end-of-life care was measured by questionnaires to relatives, discharging doctors and nurses, and municipality health staff. The ABPC comprised a physician checklist, instructions for medical professionals, a medication template to be personalized, an added standardized text to discharge papers, information pamphlets for patients and relatives, and a box of medicine and utensils.

***Results:*** Among the 40 included patients (mean age 84, standard deviation [SD] 7.7), four experienced improvements at home and resumed active treatment. The patients who died had an average survival time of 3.8 days (SD 7.5). According to relatives, 90% of patients died a dignified death without suffering. Municipality nurses rated the usefulness of the ABPC at 96 (interquartile range 88; 100) on a 0–100 scale, and all health care staff wanted to use the ABPC again.

***Conclusion:*** The ABPC showed great potential as a tool for discharging dying patients without specialized palliative needs to good-quality end-of-life care at home. The ABPC was widely accepted by relatives and all health staff. The ABCP is ready for large-scale testing with patient subgroups and economic analysis.

## Key Points

Why is this topic important?

With a growing elderly population, tools for providing end-of-life care are needed.

1) What does this study attempt to show?
2) The usefulness and quality of an acute basic palliation concept for discharging a dying patient for end-of-life care at home.
3) What are the key findings?
4) Relatives reported in 86% of cases that their loved one had died a dignified death without suffering. Municipality health staff performing the end-of-life care at home scored the usefulness of the concept at 89 on a 0–100 scale where 100 was the best possible, and all wanted to use the concept again.
5) How is patient care impacted?
6) Relatives experienced a very high degree of symptom relief in their loved ones and stated that they died a dignified death in the place they wanted.

## Introduction

Palliative care (PC) forms a cornerstone of patient-centered care in modern health care systems. It focuses on alleviating symptoms such as pain and stress in patients with serious and incurable illnesses. A consensus-based definition is: “Palliative care is the active, holistic care of individuals across all ages with serious health-related suffering due to severe illness and especially of those near the end of life. It aims to improve the quality of life of patients, their families, and their caregivers.”^1^ As the global population ages, the demand for PC services is rising. In Denmark and most other countries, PC is offered at two levels: specialized and basic.^2^ Specialized PC is for patients with complex palliative needs; it is structured, follows guidelines, and is delivered by specialists in hospices, hospitals, or in the patient’s own home. Specialized PC is provided to 18% of dying Danes.^3,4^ It requires referral and evaluation if the patient is complex enough for specialized PC. This is a process of days to weeks before the patient is offered specialized care. The remaining 82% of dying Danes receive basic PC provided by health care professionals without an education in palliation, e.g., in emergency departments (EDs).^3,4^ Basic PC is often unstructured and lacks standardized approaches.^5^ If a patient becomes terminally ill, acutely demanding end-of-life care immediately, this can never be delivered by the specialized palliation in Denmark. These terminal patients, due to e.g., a bleeding in the brain, an infection in a severely chronically ill citizen, or an abdominal catastrophe with no possible cure, rely on end-of-life care by any available doctor in the current specialty. All hospital treatment, home care, and treatment by general practitioners are free for all in Denmark and financed through taxes. End-of-life care, or terminal care, in this article is defined as palliation in the last days to few weeks of a patient’s life.

Most Danish citizens prefer to pass away in the comfort of their own homes but are often brought to the ED close to their death.^6–8^ The ED is not an optimal place for end-of-life care.^9^ Nevertheless, a Danish study found that 86% of ED staff opted to admit patients for end-of-life care rather than sending them home due to logistical and professional challenges.^10^ In this study, we attempted to reduce the professional and logistical challenges of discharging a patient for end-of-life care from an ED if they did not have specialized palliative needs. This was done using the acute basic palliation concept (ABPC), which was developed in our group and described previously in a pilot study. The ABPC was developed as a tool to discharge dying patients with a very short life expectancy (days) to die with high-quality end-of-life care in their own home. The ABPC included instructions for all health care (hospital and municipality doctors, nurses, and helpers), a doctor checklist of mandatory tasks, information material for patients and their loved ones, as well as a box with enough medication and utensils needed by 95% of these terminal patients. This has previously been established by counting what had been used from the first version of the collected boxes in the pilot study (boxes were collected after the paints died).” The aims of this study were (1) to measure the percentage of relatives who responded that the dying person had a dignified death and (2) to measure the percentage of health care staff involved with the patient’s end-of-life care who rated the ABPC as a good tool for providing end-of-life care.

## Material and Methods

This observational prospective cohort study took place in the ED of Aalborg University Hospital, Denmark. Approval for the study was granted by the hospital administration (K2022-030), and the regional ethical committee of Northern Denmark exempted the need for further approval, as this was a quality improvement project with reference number 2022-000764. In Denmark, a quality improvement study requires oral but not written consent, and this was obtained. The study was reported following the strengthening the reporting of observational studies in epidemiology guideline.^11^ In Denmark, there is a specialized offer of PC for patients who typically have a complex or long-term palliative course, while patients with a sudden need for PC receive treatment from general practitioners or hospital doctors without palliative training. This latter group makes up about 80% of patients and is the group the ABPC is targeted toward.

Patients were included consecutively. A total of 40 dying patients were discharged to receive end-of-life care using the ABPC in their own home. Inclusion criteria encompassed individuals aged 18 years and older who were being discharged for end-of-life care and who were actively dying (i.e., terminal) as evaluated by a senior physician in the ED. Exclusion criteria included clinical conditions that were not suitable for home-based palliation in the opinion of the municipality nurse or the hospital doctors or nurses (e.g., extensive vomiting or bleeding), cases where the patient’s residence was unsuitable (e.g., rat infestation), and patients who wanted to die at the hospital.

A collaboration in the North Denmark Region involving two PC departments, two EDs, and the patient organization “DanAge Organization” formed the ABPC. The concept was tested against standard practice in a pilot study.^12^ The experiences from the pilot study formed the basis for the current version of the concept used in this study.

The ABPC comprised the following components: a physical box that on the outside carried a physician checklist inspired by the principles from the aviation industry (Appendix A). The box included Instructions for municipality nurses, a medication decision tool designed for use by nurses, information pamphlets for the patients and relatives (a one-pager and a folder of eight pages in the box), and enough medicine/utensils to cover the needs for 95% of patients as listed in Table 1. This has previously been established by counting what had been used from the first version of the collected boxes in the pilot study (boxes were collected after the paints died). In addition, the concept consisted of electronic instructions for doctors, a medications template in the electronic patient chart, and a standardized text added to the discharge papers for use in the patient’s home containing legal information relevant to end-of-life care. The medication template consisted of subcutaneously administered Morphine (5–10 mg up to 24 times/24 hours), Midazolam (2.5 mg up to 24 times/24 hours), Haloperidol (1.25 mg up to 10 times/24 hours), and Glycopyrronium (0.2 mg up to 6 times/24 hours) when needed. For the treatment of constipation, suppositories with Bisacodyl (10 mg up to 2 times/24 hours) were included. The maximum number of doses could be increased by a doctor if needed. The concept box was packed by the hospital pharmacy, and the production cost was approximately 100 euros. All boxes were packed the same and not changed for individuals. This meant that all patients were prescribed morphine, midazolam, haloperidol, glycopyrronium and bisacodyle on demand. However, the doses of each drug were fitted to each individual patient (one of the checkpoints at the checklist).

**Table 1.** The Content of the Acute Palliation Concept’s Box

Quantity	Medication, utensils, and information material	Median use (Q1; Q3)	95% of patients need covered by
20 pcs	Morphine *10 mg/ml—1 ml per ampoule*	4 (2; 10)	20
20 pcs	Midazolam *5 mg/ml—1 ml per ampoule*	2 (1; 6)	14
5 pcs	Serenase *5 mg/ml—1 ml per ampoule*	0 (0; 0)	2
5 pcs	Robinul *0,2 mg/ml—1 ml per ampoule*	0 (0; 1)	4
6 pcs	Dulcolax suppositories, *10 mg*	0 (0; 0)	6
15 pcs	Natrium chloride *9 mg/ml—20 ml per ampoule*	2 (1; 6)	15
3 pcs	Subcutaneous needles	0 (0; 2)	3
10 pcs	Caps for subcutaneous needles	3 (0; 10)	10
40 pcs	1 ml syringes	10 (4; 18)	40
10 pcs	5 ml syringes	2 (1; 6)	10
30 pcs	Filter cannulas	11 (5; 19)	30
100 pcs	Swabs	11 (1.5; 62)	100
3 pcs	Fixation plasters	0 (0; 3)	3

Written material on the cover or in the concept box	
1	Checklist to be completed by the discharging doctor (22 tasks)
1	Short written information for patients and relatives (1 page)
1	Long written information for patients and relatives (8 pages)
1	Written instructions for municipal caregivers about end-of-life care (10 pages)
1	Decision tool for medication administration for municipality care givers (flowchart)
1	Discharge papers with the treatment plan including “do not resuscitate”.

**FIG. 1.**
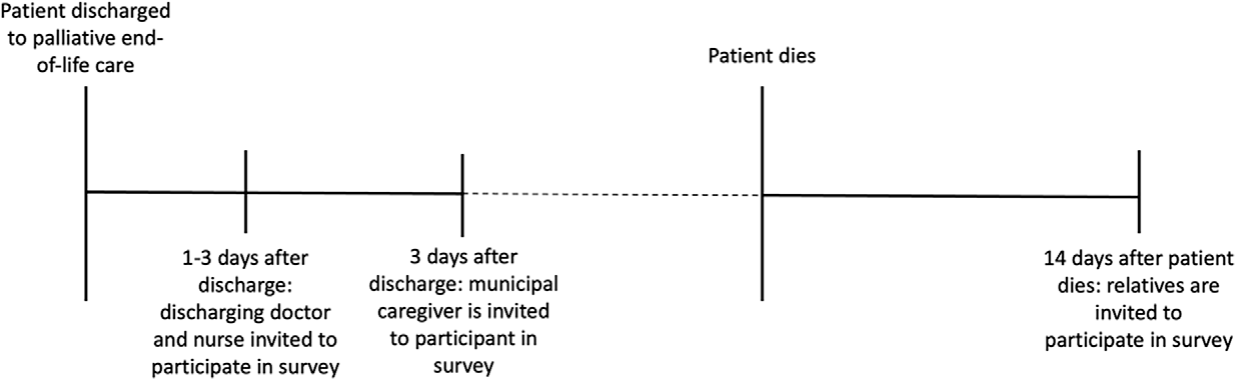
Data Collection Timeline.

Figure 1 illustrates a timeline for data collection. Patients who were dying were treated according to the ABPC and discharged. All health care services, also the ABPC, including medicine and medical supplies, are free for all Danish citizens. Demographic information about the patients was obtained from the electronic medical charts. Nurses and doctors discharging the patient received electronic questionnaires within 48 hours. The municipality health staff were sent electronic questionnaires at least one day after the patient had arrived home or at the nursing home. Relatives were contacted no earlier than past the funeral; this happens within two weeks in Denmark. Two relatives per patient were invited to participate in the study.

## Description of Questionnaires

No standardized validated questionnaires exist for this area; therefore, they were developed for the pilot study.^12^ The questionnaires were tested among health care personnel before the pilot study. The questionnaires were then adjusted from the free-text field in the pilot study and qualitative interviews with relatives and health staff (data not yet submitted) before use in the current study. Questions were either answered dichotomously with “yes” or “no” or on a 5-point Likert scale with the following options: “to a very low degree,” “to a low degree,” “to some degree,” “to a high degree,” or “to a very high degree.” Furthermore, all questions had an “I don’t know” option. The options “to a high degree” and “to a very high degree” were considered a positive response. The remaining answers, including “to some degree,” were considered negative. The effectiveness of the ABPC was scored on a 0–100 visual analogue scale with “worst possible” at 0 and “perfect” at 100.

The questionnaire for relatives included questions on their perception of symptom management, feelings of safety with the end-of-life care, ease of understanding information, and involvement in decision-making. Questionnaires for health care professionals covered past experiences, information delivery, and patient/relative involvement in decision-making. Additional municipal caregivers were questioned about symptom management and overall professional confidence in providing end-of-life care. Municipality caregivers were primarily nurses but also certified health care assistants (Danish “SOSA” with two years of education in assisting nurses in care and medication administration). Discharging doctors reported their confidence in the selection of end-of-life medication, the discharge process, and familiarity with necessary documents. And discharging nurses reported their confidence in end-of-life patient discharge. All health care professionals were asked to estimate their time used when providing end-of-life care.

## Data Collection and Analysis

Patients were included from May to December 2023. Data were gathered and organized in REDCap, an electronic data capture tool.^13,14^ Data management and statistical analysis were conducted using SAS Enterprise Guide 7.1 (SAS Institute Inc., Cary, NC, USA). The results of the questionnaires were displayed as a percentage of positive responses out of all responses and presented in spider plots. Numeric data were expressed as either the median and interquartile range or the mean and standard deviation, depending on data distribution.

## Results

The demographics for the included patients are shown in Table 2. The patients included were old, cognitively impaired, or severely disabled prior to admission. None of the patients were readmitted, but four improved substantially, and active treatment was started again; three patients were still alive when the study ended. The mean survival time after discharge was 3.8 (7.5) days.

**Table 2.** Demographics for the 40 Patients Discharged with the “Acute Basic Palliation Concept”

Patients	*N* = 40
Age: mean (SD), years at discharge	84 (7.7)
Sex, % men (*n*)	45 (23)
Time to death: mean (SD), days from discharge^b^^,^^c^	3.8 (7.5)
Patients not able to walk before admission^a^ % (*n*)	77 (30)
Dementia or known cognitive impairment: % (*n*)	60 (24)
Cancer: % (*n*)	28 (12)
Admission facilitated by Emergency medical services: % of patient group, (*n)*	82 (37)
Admitted from: % of patient group, (*n*)	
Own home	45 (18)
Nursing home	55 (22)
Other	0 (0)
Regular medication usage prior to inclusion: % of patient group, (*n*): Morphine	34 (16)
Benzodiazepine	8.9 (4)

SD, standard deviation, IQR; 25 to 75 interquartile range.

^a^ Either confined to bed, used lift or wheelchair, or activities of daily living was described ‘low’ without additional details in the medical record.

^b^ Not counting the admission that resulted in inclusion the study.

^c^ Two patients were still alive in the acute palliation concept group 60 days after discharge.

A total of 40 relatives (representing 34 out of the 37 patients who died) participated. Two couples were allowed to answer together (husband and wife). Of the invited staff, answers were returned by 78% (31/40) of the municipality health workers, 78% (31/40) of hospital doctors, and 50% (20/40) of hospital nurses. Nonresponders at the hospital included staff that had answered previously or did not want to participate due to a high workload. We were not able to contact the municipality health staff that did not answer.

### Objective 1—patients passed away in a dignified way without suffering

**FIG. 2.**
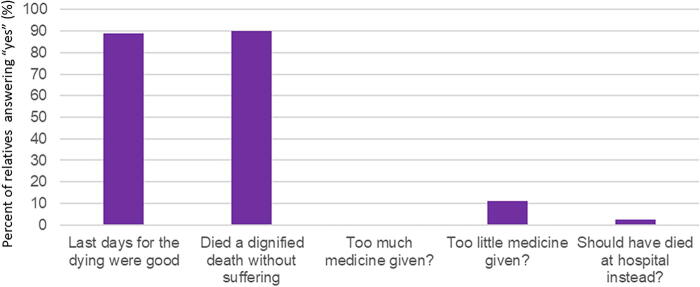
Relatives view of the end-of-life care for the dying. Relatives were invited to participate in a questionnaire after the patient had died and been buried. The graph below showed how many of the patients’ loved ones that answered “Yes” to the questions at the X-axis.

Figure 2 shows results from the 40 relatives’ perspectives on the end-of-life care quality for the patients (95% first-degree relatives): The primary aim was met in 89% of patients (*n* = 34), and 89% also answered that their relative’s last days were good (*n* = 33). Seven (19%) of the patients had been brought to the hospital against their will. None of the 40 relatives felt that the patient had received too much medicine, but 11% (*n* = 4) felt insufficient amounts of medicine had been administered. Poor or insufficient treatment of pain was rarely reported (Fig. 2).

### Objective 2—Health care staff found the ABPC useful and wanted it permanently implemented

**FIG. 3.**
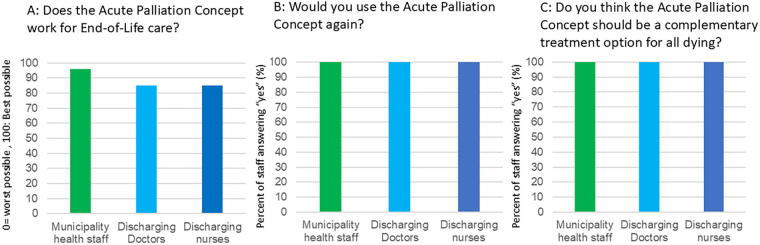
The usefulness of the acute palliation concept evaluated by the health care staff using it for end-of-life care in dying patients without specialized palliative needs.

Figure 3 shows the health care staff at the hospital (*n* = 51) and in the municipalities (*n* = 31) evaluation of the ABPC. Highest scores were seen among municipality health staff (96%). All doctors and nurses wanted to use the ABPC again and recommended it to become a free and permanent tool in end-of-life care for all relevant patients.

**FIG. 4.**
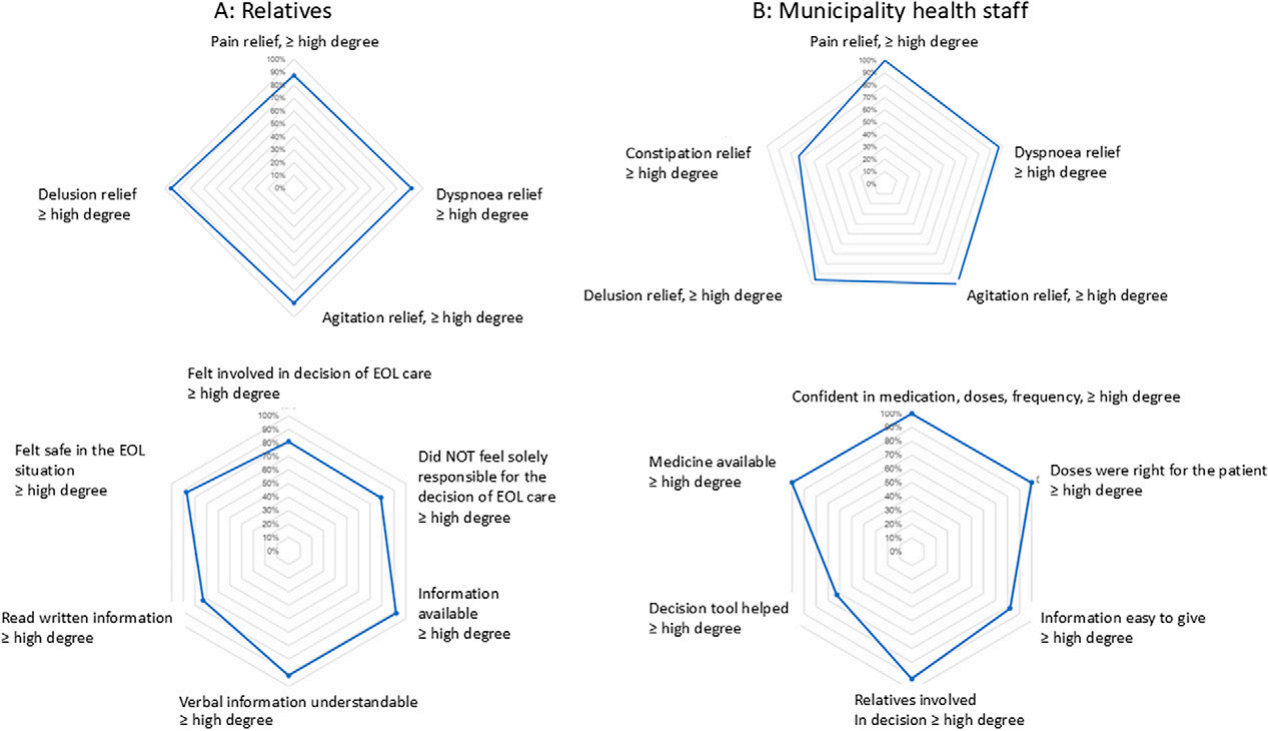
Symptom-relief was achieved for all symptoms in most patients.

Figure 4A shows symptom relief evaluated by relatives and municipality health staff. Insufficient symptom relief reported by municipality health staff was rare (Fig. 4B). None of the municipality health staff thought the medicine doses were too high.

### Doctors and nurses supported by the ABPC in end-of-life care

**FIG. 5.**
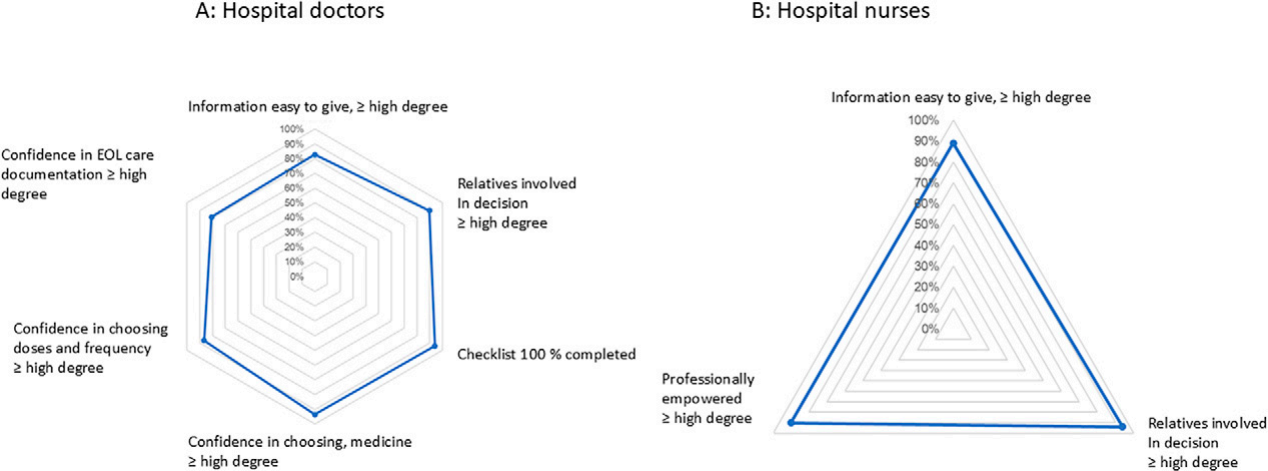
Percentage of responding hospital staff answering, “to a high degree” or “to a very high degree” (31 doctors, 20 nurses).

As seen in Figure 5, the performance scores for the ABPC were high. This was despite the low level of experience of hospital staff: 56% of doctors and 60% of hospital nurses had performed end-of-life care less than six times. The municipality staff were more experienced (13% had performed end-of-life care less than six times). The time spent initiating end-of-life care by the doctors in the hospital was a median of 1.5 hours (1; 1.5). The doctor checklist completion rate was 93% (*n* = 38).

### Medicine and utensils used

Seventeen boxes were available for pickup; the rest were discarded by the municipality employees according to local practice. The used materials can be seen in Table 1.

## Discussion

This observational cohort study measured the relatives and caregivers’ perceptions of the ABPC for providing end-of-life care in 40 Danish patients. They were brought to the emergency department actively dying and discharged to their home for end-of-life care.

### Aim 1—patient died a dignified death without suffering

Relatives stated that most patients had experienced a dignified passing without significant suffering and that their last days were positive. More than half of the patients had severe cognitive impairment prior to hospitalization and were not able to be involved in the decision about whether to stop treatment or where to spend their last days. This was the rationale for having “family member involvement” as a checklist item. This was also the justification for selecting family members’ opinions of the end-of-life care as the primary outcome. A study by Wendler et al. documented that two out of three patients wanted their relatives to decide their treatment if they experienced an event of decisional incapacity.^15^ This has been followed up by studies and recommendations that clarify the importance of shared decision-making involving relatives and interdisciplinary partners.^16–18^ The checklist item as described above was included to ensure this, and the questionnaires showed a high level of effectiveness.

The comprehensive information materials were created in collaboration with a patient organization (Dan-Age), which might explain the high score for understanding information as measured among family members. A previous review suggested the following indicators for good end-of-life care communication: clear, sensitive, honest, respectful, open, transparent, consistent, and open for discussion over time.^19^ Most of these qualities were reflected in the written material. Furthermore, most relatives did not know what they should expect and how they should behave. They liked to be informed by the written information material, e.g., not push the patient to drink or eat when they did not want to.

Good management of symptoms is an important part of end-of-life care.^19^ However, most studies of symptom management have been performed in patients under specialized PC, making it difficult to compare results meaningfully.

The medication template in the ABPC was chosen to empower doctors with a framework of drug types and doses from which they could start tailoring treatment to the specific patient. The selection of drugs in the ABPC aligned with previous studies of end-of-life care in Eds.^20^ For safety reasons, there was a checklist item of “individualization of doses,” and all doctors were supervised by an experienced doctor. Doses needed can be high as patients transitioning to end-of-life care in EDs often present with severe symptoms. The pilot study of the ABPC indicated that patients palliated with the ABPC did not die sooner compared with standard treatment (2.5 days versus 2.0 days) (under review in the Danish Medical Journal). This reflects previous studies of end-of-life care showing that good palliation does not result in an earlier death.^12,21^ The percentages of patients in the current study who were insufficiently treated for pain, dyspnea, agitation, or discomfort were very low (6%–11%) compared with other studies of nonspecialized palliation.^6,20,22^ There is still room for improvement, as 12% of family members felt that insufficient amounts of medicine had been given. Most patients in the study had been very frail for months or years; hence, a palliation plan prior to acute deterioration would have been advantageous. This was underlined by the fact that seven patients had been brought to the hospital against their will, due to severe symptoms that could not be handled at home for different reasons (difficulties in finding sufficient documentation of “do not resuscitate” in the home, municipality staff insecurity/inexperience, difficulties getting in contact with a general practitioner, etc.). This is an unfortunate situation for the patient who wants to pass away in the comfort of home and for the relatives who are aware of this. Besides being frustrating for relatives that try to prevent the hospital visit, it is expensive for society. We have started to address this problem in our region, and due to the complexity, it will take time to change. The ABPC might be a part of solving this issue.

### Aim 2: the ABPC was useful for health care staff who wanted it implemented permanently

All doctors and nurses wanted to use the standardized ABPC again and thought the ABPC should be free for the patient and a permanent tool in end-of-life care for all patients. This finding supports a U.S. study including patients who were comparable to the present study regarding age, frailty, and cognitive impairment. Chor et al. documented that structured guidelines provided qualified assistance for health care professionals in the ED.^21^ In our study, the structured ABPC scored very high for use by the municipality staff treating the patients and solved many of the previous known problems in sector transition, including measures to avoid bedsores.

### The ABPC still worked well despite inexperienced hospital staff

In the current study, we found that many hospital doctors and nurses were inexperienced in palliation, in contrast to the municipality employees. The time spent starting end-of-life care by the doctors in the hospital was a median of 1.5 hours (1; 1.5), which is manageable in the Department of Emergency Medicine, and there was a high degree of checklist completion. Based on the pilot study, we knew that junior doctors find documentation particularly difficult.^12^ This is where the checklist items especially support inexperienced staff. Further, it is well known that it is challenging for nonspecialist nurses to care for palliative patients.^23^ This was the basis for the extensive material in the box for the nurses, which was built on principles of providing care for dying patients. It was revised after the pilot study as family member interviews identified that some municipality staff described a “death battle” at the very end of life (a patient in obvious distress). This was described as “normal” and used as a cause for not administering medicine for symptom relief. The written instructions in the present study explicitly addressed this practice as unacceptable, emphasizing that treatment for symptom relief should persist until the patient has passed away. This may have influenced the improvement in the results for symptom management compared to the pilot study.

### Strengths and limitations

The study demonstrated notable strengths. Firstly, a key strength was the involvement and evaluation of most stakeholders in the end-of-life care, such as family members, municipality health staff, and hospital nurses and doctors. This comprehensive approach ensured thorough documentation of patient trajectories across the different sectors. In the northern Denmark region, obtaining medication for end-of-life care can be challenging, especially on weekends when the nearest open pharmacy may be up to 150 km away. In addition, family members are required to pick up and pay for the medication upfront after a standard discharge for end-of-life care, with reimbursement occurring later. This frequently leads to hospital admissions if the family is unable to provide this support. Therefore, including a complementary medication box at the ED was a strength of the concept. A strength is also that Danish health care services are free for everyone, including ABPC, with medicine and medical supplies included, making this service equally accessible to all regardless of the financial status of the patient.

The adaptation of the concept and questionnaires after the pilot study results represented another strength, and the result confirmed and strengthened the results from the pilot study. The retrieval of the concept’s boxes allowed a good estimation of the medication requirements for the cohort. The recommendation of specific drugs and doses supplemented by a checklist for individualized adjustments was another strength.

However, several limitations must be acknowledged. The use of unvalidated questionnaires, though necessitated by the absence of standardized alternatives, poses a primary limitation. Generalizability is restricted to patients in EDs since all patients were hospitalized due to symptoms deemed unmanageable at home. In addition, the study’s applicability is limited to patients with a short life expectancy rather than those with weeks to months. The study would have been stronger as a randomized controlled trial; however, this is not ethically feasible with such an extremely vulnerable group. The sample size, while deemed adequate for evaluating the concept’s efficacy in an ED, was insufficient for subgroup analyses. Nonresponse bias and lack of information about nonresponding participants present additional limitations. Detailed information about, e.g., functional ability, mental status other than reported in the table, and whether the patients were eating or drinking was not collected because it was not possible due to the patients’ condition and fragility. The information could have been collected if information from the municipality had been available, but this is not the case in this type of quality study in Denmark. Qualitative interviews are currently underway to complement the study’s findings by exploring the experiences of staff and relatives.

## Conclusion

In this observational study, the ABPC showed great potential as a tool for discharging patients from EDs for high-quality end-of-life care at home. The ABPC was positively evaluated by the relatives and widely accepted by all 82 health staff members. The findings require confirmation in larger studies with related analysis of health economics as well as qualitative interviews.

## Declaration of Interest

None of the authors have any competing interests to declare.

## Abbreviations Used

ABPC: acute basic palliation concept
ED: emergency department
IQR: interquartile range
PC: palliative care
SD: standard deviation

## Appendix A

### Patient name and CPR number

When the box is no longer needed, we will collect it within a few days. Place any unused medication and utensils in the box. Email the address details to apslk@rn.dk.

**Table A1.** Physician check list for the Acute Basic Palliation Concept with box: Palliative care in the final moments at home

Check X	Doctor's responsibilities - Do not send the box with the patient until all items on the checklist are completed.
	**Send an E-mail to**projectresponsible@rn.dk with the patient’s name and CPR number
	**Ensure the patient has an expected remaining lifespan of only a few days to a few weeks.** **If this is not the case, seek an alternative solution**
	**The patient and relatives (at least 2) have been involved and informed that the patient is dying and transitioning to a terminal phase.** Names of the two relatives in the discharge summary/medical record
	**Relatives informed that they will be contacted by phone a few weeks after the patient's death to follow up on the last period** (participation is voluntary)
	**Distribution of informational materials from the box to relatives** while they are in the Emergency Department
	**Medicine list reviewed**: Remove all non-palliative medicine.
	**“Acute Basic Palliation Medicine package”** prescribed in the electronic medical file.
	**Adjust doses** if needed (e.g. Morphine dose HAS to be at least 1/6 of the patient’s previous daily dose)
	**Transfer the medicine list to the electronic “Shared Medical Record”** (available for all health staff regardless of sector)
	**Prescriptions made of** 10 ampoules of each medication on the medicine list.
	**Red discharge summary for the general practitioner made.** Dictation: “insert standard discharge summary for Acute Basic Palliation Concept (standard text 022)”. Add a summary of the course of events and any corrections to medication, if applicable. See standard text 022 at the website PRI.rn.dk number “34949“
	**Red discharge summary for the general practitioner printed, signed and put in the concept box.** Go to the secretariat and ask a secretary to write it immediately.
	**Application for free medication made electronically** (see PRI.rn.dk “Terminal care subsidy”)
	**Correct doses in the flow chart** in the box so they are identical with the Shared Medical Record
	**Sign the flow chart.**
	**The municipality health staff, which receives the patient, informed by phone** (call the nursing home if the patient resides there) and simultaneously obtain the phone number of the nurse to be contacted in the next checklist item
	**Municipality nurse contacted by phone.** Information about the patient's habitual condition is confirmed, and the treatment plan is initiated and handed over.
	**Insert 2 subcutaneous needles.**
	**Is the patient receiving oxygen? Create a discontinuation plan with the attending physician and inform the receiving home care nurse + nurse** (typically: 10 mg subcutaneous morphine approximately 15 minutes before arrival at home. Discontinue oxygen 15 minutes after morphine has been given).
	**Inform the patient’s general practitioner by phone** if possible (every weekday during daytime hours (8AM-4PM)
	**Ensure Ambulance services are ordered and “no resuscitate” orders are w**ritten or orally conveyed.
	**Medical treatment responsibility for 72 hours:** Discharging physician name:_____________phone:____________until date / 202_ time _____ thereafter: Call 97 66 00 09 and ask for the on-call Emergency Physician (kode number____)
	**Label with patient name and CPR-number to the Emergency Physician:** Label on the list in the control center and orally conveyed to the on-call Emergency Physician that there is “72 hours responsibility” for the pt.

Quality project from Aalborg University Hospital and North Jutland Regional Hospital, Hjørring. The content of the box has been developed in collaboration with the palliative teams and emergency departments from both hospitals, as well as the Ældre Sagen Nordjylland (DanAge organization)
